# Implementation of gatekeeper training programs for suicide prevention in Japan: a systematic review

**DOI:** 10.1186/s13033-018-0258-3

**Published:** 2019-01-03

**Authors:** Naohiro Yonemoto, Yoshitaka Kawashima, Kaori Endo, Mitsuhiko Yamada

**Affiliations:** Department of Psychopharmacology, National Center of Neurology and Psychiatry, 4-1-1 Ogawahigashi, Kodaira, Tokyo Japan

**Keywords:** Suicide prevention, National policy, Gatekeeper program, Implementation, Japan

## Abstract

**Background:**

Suicide is a critical global health issue. Japan has had a high suicide rate for the last 12 decades. In 2007, the Japanese Central Government Office issued the “General Principles of Suicide Prevention Policy”. An important component of this policy was the gatekeeper training (GKT) program. GKT is a widely recommended suicide prevention intervention. This study aimed to investigate the association between the announcement of the national suicide prevention policy and implementation of GKT programs in Japan.

**Methods:**

We performed a systematic review of public documents from central and local governments and research literature using three Japanese databases and PubMed. Characteristics of eligible reports and the report quality of local government information were summarized.

**Results:**

All local governments provided information about GKT activities. Over 80% of local governments had specific GKT webpages, but useful localized information and program evaluations were limited. Our literature search identified 122 eligible reports. The number of reports increased markedly from 2011 to 2014. However, few of the reviewed research studies used validated outcome measures.

**Conclusions:**

The announcement of the national suicide prevention policy increased the implementation of GKT programs in Japan. However, there remains a need for integration of knowledge and evaluation of GKT programs.

**Electronic supplementary material:**

The online version of this article (10.1186/s13033-018-0258-3) contains supplementary material, which is available to authorized users.

## Background

Suicide is a critical global health issue. Japan is a developed country and has had a high suicide rate for the last 12 decades. The suicide rate per 100,000 people remained high following a marked nationwide increase in 1998 (suicide rate 26.1) that lasted until 2011. Although the suicide rate decreased after 2011, it was still high (16.7 per 100,000 people) in 2017. Therefore, suicide remains a priority mental health issue in Japan [1].

A systematic review by Mann et al. [2] included gatekeeper training (GKT) programs as one of five recommended suicide prevention strategies. The term “gatekeeper” refers to people who have primary contact with individuals at risk for suicide, and who can identify such individuals by recognizing suicidal risk factors. GKT “teaches specific groups of people to identify people at high risk for suicide and then to refer those people for treatment” [3]. GKT is often integrated into suicide prevention strategies aimed at educating social and community facilitators to identify signs of suicidal behavior and refer individuals to appropriate services [4].

In some countries, suicide prevention strategies and activities focus on increasing access to mental health services for vulnerable people via general practitioners. In contrast, prevention strategies in Japan are unique in that they emphasize public awareness of suicide and social and economic factors related to suicide prevention. Japan has adopted a comprehensive approach to suicide prevention that involves healthcare and non-healthcare sectors [5].

In 2007, the Japanese Cabinet Office released the “General Principles of Suicide Prevention Policy”. This national policy recommended the use of GKT programs for suicide prevention, as these programs can be integrated into existing strategies. The policy suggested that GKT programs should be provided to various groups, such as general physicians, teachers, public health nurses, long-term care support specialists, local welfare commissioners, child welfare volunteers, and local public health officers. Evidence suggests that GKT programs for suicide prevention are effective for increasing knowledge, building skills, and molding trainees’ attitudes. GKT programs have been implemented and tested with several different populations, including schools and minority groups [3]. Implementation of GKT has been led by Japan’s Ministry of Health, Labour and Welfare (MHLW), with local authorities expected to implement and be primary coordinators for GKT programs. A special fund is available to support this function. Previous studies have reported the impact of the policy on overall suicide rates [5–8]. This study aimed to investigate the association between the announcement of the national suicide prevention policy and implementation of GKT programs in Japan.

## Methods

We were unable to find a comprehensive registered database or list of activities about GKT in Japan; therefore, we used two methods to systematically review GKT implementation. First, we systematically searched public documents from central and local governments for any mention of GKT [9, 10]. This was because local authorities are responsible for planning and conducting GKT programs based on the national suicide prevention policy. These documents covered 47 prefectures and 20 major designated Japanese cities with high populations and other public resources. We also examined and evaluated websites and published documents linked to central and local government and other public institutions, such as non-governmental organizations (NGOs), for information about GKT [10, 11]. Most information was collected from public domain websites and Google searches. The search terms were “suicide”, “gatekeeper”, or the name of the local government. If necessary, we contacted institutions to obtain information. Information was accessed up to July 2018. We evaluated the information collected using eight criteria developed for this study: (1) specific webpages, (2) leaflets developed by local government, (3) training textbooks and materials developed by local government, (4) additional materials developed by local government, (5) local government-registered activities, (6) local government activity notices, (7) local government activity case reports, and (8) evaluations of these activities. We calculated the frequencies of the types of information provided by prefectures and major designated cities. Table 1 shows the characteristics of the information provided on GKT.

**Table 1:** Characteristics of information on GKT provided by local governments (prefecture n = 47, designated city n = 20)

	n (%)
*(1) Specific local government webpages on GKT*	
Prefecture	38 (81.0)
Designated city	16 (80.0)
*(2) Leaflet developed by the local government*	
Prefecture	15 (31.9)
Designated city	3 (15.0)
*(3) Training textbook and materials developed by the local government*	
Prefecture	12 (25.5)
Designated city	2 (10.0)
*(4) Additional materials developed by the local government*	
Prefecture	15 (31.9)
Designated city	5 (10.6)
*(5) Local government registered activities*	
Prefecture	4 (8.5)
Designated city	0 (0.0)
*(6) Local government activity notices*	
Prefecture	46 (100)
Designated city	20 (100)
*(7) Sharing of activity case reports by the local government*	
Prefecture	5 (10.6)
Designated city	0 (0.0)
*(8) Local government evaluation of activities*	
Prefecture	8 (17.0)
Designated city	0 (0.0)
*(9) Central government case reports of local activities*	
Prefecture	14 (29.8)
Designated city	0 (0.0)

**Fig. 1:**
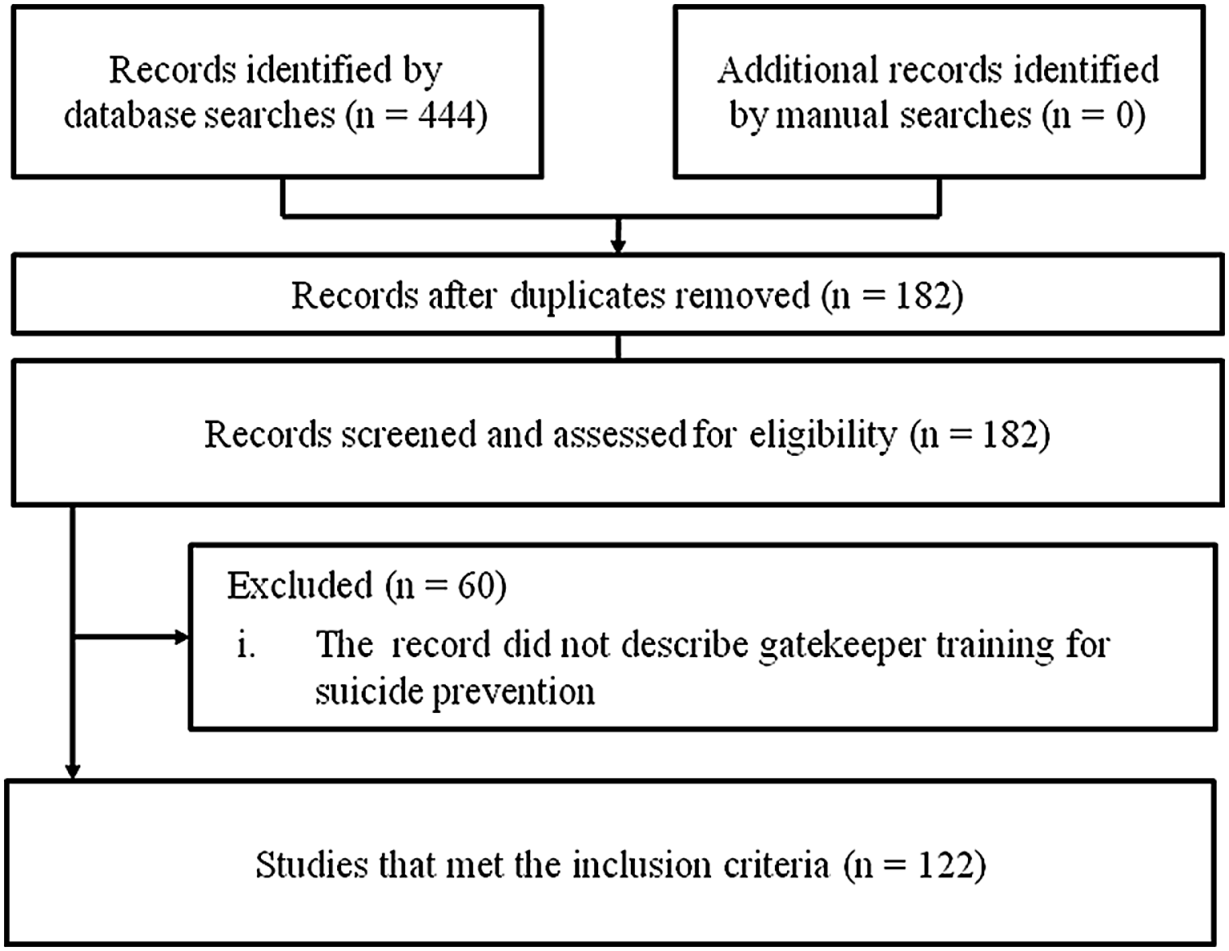
Flow chart of the literature search

**Fig. 2:**
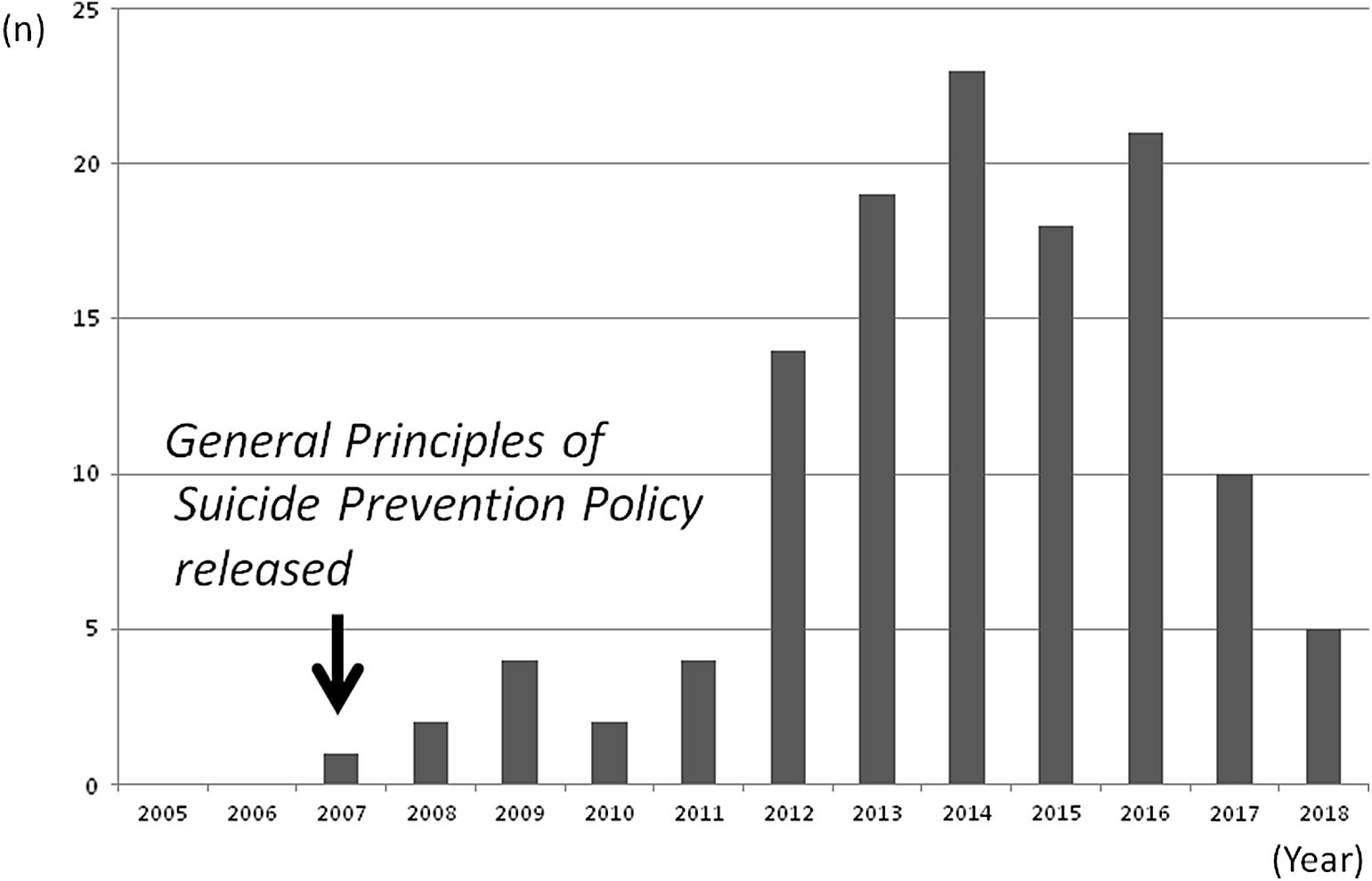
Trends in reported frequency of information provided

Second, we searched for articles on GKT up to July 2018 using three Japanese literature databases: Ichushi, a Japanese medical journal database (http://www.jamas.or.jp/); Cinii, a database of academic information about articles, books, journals, and dissertations in Japan (https://ci.nii.ac.jp/); and Google Scholar in Japanese. We also searched PubMed in English. We considered articles that represented an index of the implementation of GKT programs. The English keywords and formulae used in the search were: (suicid*) OR (self-harm*) OR (selfharm*) OR (self-poison*) OR (selfpoison*) OR (overdose*) OR (over-dose*) OR (self-injur*) OR (selfinjur*) OR (self-mutilation*) OR (selfmutilation*) OR (automutilation*) OR (auto-mutilation*) OR (self-destructive*) OR (selfdestructive*) AND (gatekeeper*) AND Japan. The Japanese keywords and formulae used were: (jisatsu) OR (jishou) OR (jikohakai koudou) OR (jison) OR (kishinenryo) AND (gatekeeper). Eligible criteria were reports on any activities and evaluations of GKT programs for suicide prevention in Japan that contained “gatekeeper” and related terms. All types of reports were eligible, including conference and review papers. We classified report characteristics and calculated frequencies and percentages of the information provided (Table 2, Figs. 1, 2). This review was reported in accordance with Prefered Reporting Items for Systematic Reviews and Meta-Analyses statement (Additional file 1).

**Table 2:** Characteristics of publications on GKT (N = 122)

	n (%)
*Target population*	
Lay persons	14 (11.5)
Public sector workers	27 (22.1)
Private sector workers	3 (2.5)
Schools	16 (13.1)
Clinics and hospitals	6 (4.9)
Health centers	12 (9.8)
Pharmacies	24 (19.7)
Internet and SNS users	6 (4.9)
Other	14 (11.5)
*Report type*	
Review	54 (44.2)
Original article	28 (23.0)
Conference paper	40 (32.8)
*Publication language*	
Japanese	117 (95.9)
English	5 (4.1)

## Results

### Central government information

The website search identified GKT webpages by Japan’s MHLW that provided a sample handbook, a sample textbook on training, and video lectures. The relevant central government webpages also provided the names of staff members who offered lectures on GKT. From these websites, we extracted 228 case series reports of local government activities related to suicide prevention from 2012 to 2015 and 31 eligible cases of GKT from 14 prefectures.

### Local government information

Our review identified different types of information on GKT provided by local governments (Table 1). All local governments had information about GKT activities, and over 80% had specific webpages about GKT. Useful and localized information was provided by 15 prefectures (31.9%) and three cities (15.0%), which included a sample of a leaflet developed by the local government. In addition, 12 prefectures (25.5%) and two cities (10.0%) provided a sample of a training textbook developed by the local government, and 15 prefectures (31.9%) and five cities (10.6%) provided additional materials developed by local government. Unfortunately, few local government activity and evaluation reports were available. We also found that more than 20 NGOs conducted GKT. These NGOs provided GKT programs and sometimes collaborated with local governments. Some local governments contributed financial resources to NGO GKT programs.

### Published research

The literature search identified 424 studies: Ichushi n = 115, Cinii n = 51, Google Scholar (in Japanese) n = 245, and PubMed (in English) n = 13. There were 122 eligible studies after removing duplicates and excluding those that did not discuss GKT for suicide prevention (Fig. 1). The target populations of the reviewed studies varied (as indicated in the national suicide prevention policy): public sector workers, private sector workers, bar keepers, pharmacists, teachers, university officers, general physicians, nurses, mental health providers, and Internet and SNS users (Table 2). A variety of programs were conducted to train anyone who was able to recognize and refer someone at risk of suicide. Most programs had been developed based on MHLW information. The number of eligible studies increased between 2011 and 2014 (Fig. 2). Most were reports of activities rather than comparison studies that evaluated outcomes. Few studies used validated Japanese outcome measures, such as the Suicide Intervention Response Inventory [12], the Attitudes toward Suicide Scale [13], or the Gatekeeper Self-Efficacy Scale [14].

## Discussion

### Findings

Our study demonstrated that the announcement of a national suicide prevention policy increased the implementation of GKT programs in Japan. The policy announcement was strongly associated with the number of publications reporting GKT programs. This suggests that the policy announcement led to positive changes in GKT implementation; however, there was little evidence of the integration of knowledge and evaluation of programs.

### Implementation status and related issues

There is substantial political support for GKT implementation. In Japan, suicide prevention was promoted by a basic act for suicide prevention in 2006, a policy in 2007 outlining general principles of suicide prevention, and the establishment of a special fund for local government programs. These measures led to the development of a comprehensive and multi-sector approach to suicide prevention. Their effectiveness is illustrated by the consistent decrease in the number of suicides since 2011 [5–8]. GKT is a core component of suicide prevention strategies and has been widely implemented in Japan.

Strong collaboration between the public sector and NGOs is an important part of the implementation of GKT programs, and some prefectures and cities closely collaborated with NGOs. Many suicide prevention activities were conducted by NGOs, and information about some of these activities was not available on local government websites. However, local governments provided some financial incentives and resources to support NGOs in providing GKT programs. In addition, some prefectures had a major designated city; in those prefectures, the suicide prevention role was allocated to a local authority. This is consistent with previous research that identified collaboration between NGOs and some local governments on suicide prevention in Japan [5].

Local governments provided limited information about evaluation and feedback of GKT programs. Only a small number of local government websites provided numbers of registered members, and few MHLW case report collections were provided on local government webpages. Only a few prefectures examined public awareness of GKT as part of questionnaire surveys on suicide prevention and mental health. Some research publications used pre–post designs for outcome evaluation. Although most studies reported positive effects of GKT, there was substantial variation in outcomes and some studies used ad hoc self-developed measures.

## Limitations

The study had some limitations. First, our review of websites and public documents may not have identified all GKT activities in Japan. Second, some sources may have used alternative terms for “gatekeeper” (which was one of our search terms) that were more user-friendly and attractive to the public. Additionally, some so-called GKT programs only consisted of a lecture on mental health or psychiatric information, such as a classroom lecture of 1–2 h. Other community interventions comprised multimodal programs, and it was difficult for us to isolate the GKT program (which might not have been categorized as such). Third, many programs were independently provided by small local town council offices, making it difficult to obtain a comprehensive overview of the provision of GKT programs. Fourth, some of the reports might have been misclassified, especially as some activities were multimodal or comprehensive community interventions. Fifth, we could not evaluate the outcomes of the implemented GKT programs. Our review of published scientific research found limited evidence of the short-term effects of GKT and no clear confirmation or validation of common outcomes. More studies are needed to develop databases to share knowledge and outcomes and monitor the quality of GKT programs.

## Conclusions

The announcement of a national suicide prevention policy increased the implementation of GKT programs in Japan. However, there remains a need for integration of knowledge and evaluation of GKT programs.

## Additional file

**Additional file 1.** PRISMA checklist.
